# Selection of trilateral continuums of life history strategies under food web interactions

**DOI:** 10.1038/s41598-018-22789-6

**Published:** 2018-03-14

**Authors:** Masami Fujiwara

**Affiliations:** Department of Wildlife and Fisheries Sciences, Texas A&M University, College Station, TX 77843-2258 USA

## Abstract

The study of life history strategies has a long history in ecology and evolution, but determining the underlying mechanisms driving the evolution of life history variation and its consequences for population regulation remains a major challenge. In this study, a food web model with constant environmental conditions was used to demonstrate how multi-species consumer–resource interactions (food-web interactions) can create variation in the duration of the adult stage, age of maturation, and fecundity among species. The model included three key ecological processes: size-dependent species interactions, energetics, and transition among developmental stages. Resultant patterns of life history variation were consistent with previous empirical observations of the life history strategies of aquatic organisms referred to as periodic, equilibrium, and opportunistic strategies (trilateral continuums of life history strategies). Results from the simulation model suggest that these three life history strategies can emerge from food web interactions even when abiotic environmental conditions are held constant.

## Introduction

The trilateral life history model has been proposed to describe variation in observed life history strategies among species^1^. According to the model, life history variation can be predicted by trade-offs among three variables: the age of maturation (affecting generation time), investment per offspring (affecting survival to the adult stage), and fecundity. The endpoints of these three variables are termed equilibrium, periodic, and opportunistic strategies. This life history model is an empirically derived model and considered an extension of the traditional *k*- vs. *r*-selection life history model. Because different natural resource management and biological conservation strategies are required for organisms with different life histories^2^, understanding the cause of these life history patterns is critically important.

There is a long history of studies investigating the factors that affect life history strategies in general^1,3–13^. Many of these studies have focused on the effects of environmental variation^12,14–16^, or the relative importance of environmental variation and density dependent processes^6,17,18^. For example, Winemiller and Rose^1^ attributed the causes of the trilateral life history variations to differences in physical environmental conditions experienced by organisms. However, species interactions are another important process potentially affecting the selection of life history strategies^8,19–21^. For example, life history strategies of guppies (*Poecilia reticulata*) were affected by the predators they experience^20^. Nevertheless, the importance of species interactions in the studies of life history evolution is still underappreciated. This is especially true with the studies of trilateral life history variations as most recent studies focused on the effects of physical environmental conditions^22–25^. Consequently, it is still not clear what types of life history strategies are selected when multiple stage-structured populations of different species are experiencing consumer-resource interactions.

The objective of the current study was to demonstrate the role of consumer–resource interactions in shaping the life history strategies of animal species, using a food web model consisting of multiple stage-structured populations of different species. The model was used for investigating the six properties of life history strategies of persisting species: duration of the adult stage, generation time, age of maturation, percent of offspring that survive to maturity, average fecundity, and longevity at birth (Table 1).

**Table 1:** Equations for calculating life history properties.

Descriptor	Equation
Adult Duration^1^	$\frac{({f}_{3,s}(N\ast )+{p}_{3,s}(N\ast )+m){\int }_{0}^{\infty }t{n}_{3,s}(t)dt}{({f}_{3,s}(N\ast )+{p}_{3,s}(N\ast )+m){\int }_{0}^{\infty }{n}_{3,s}(t)dt},\,{n}_{3,s}(0)=1,\,{g}_{2,s}(N\ast ){n}_{2,s}=0$
Generation Time^1,2^	$\frac{{b}_{s}(N\ast ){\int }_{0}^{\infty }t{n}_{3,s}(t)dt}{{b}_{s}({N}^{\ast }){\int }_{0}^{\infty }{n}_{3,s}(t)dt},\,{n}_{1,s}(0)=1,\,{n}_{2,s}(0)=0,\,{n}_{3,s}(0)=0$
Age of Maturation^1,2^	$\frac{{g}_{2,s}({N}^{\ast }){\int }_{0}^{\infty }t{n}_{2,s}(t)dt}{{g}_{2,s}({N}^{\ast }){\int }_{0}^{\infty }{n}_{2,s}(t)dt},\,{n}_{1,s}(0)=1,\,{n}_{2,s}(0)=0$
Percent Mature^1,2^	${g}_{2,s}({N}^{\ast }){\int }_{0}^{\infty }{n}_{2,s}(t)dt,\,{n}_{1,s}(0)=1,\,{n}_{2,s}(0)=0$
Fecundity^1^	${b}_{s}({N}^{\ast })$
Life Expectancy^1,2^	NUM/DEN_, where_ ${\rm{NUM}}=({f}_{1,s}({N}^{\ast })+{p}_{1,s}({N}^{\ast })+m){\int }_{0}^{\infty }t{n}_{1,s}(t)dt$ $+({f}_{2,s}({N}^{\ast })+{p}_{2,s}({N}^{\ast })+m){\int }_{0}^{\infty }t{n}_{2,s}(t)dt$ $+({f}_{3,s}({N}^{\ast })+{p}_{3,s}({N}^{\ast })+m){\int }_{0}^{\infty }t{n}_{3,s}(t)dt,$ ${\rm{DEN}}=({f}_{1,s}({N}^{\ast })+{p}_{1,s}({N}^{\ast })+m){\int }_{0}^{\infty }{n}_{1,s}(t)dt$ $+({f}_{2,s}({N}^{\ast })+{p}_{2,s}({N}^{\ast })+m){\int }_{0}^{\infty }{n}_{2,s}(t)dt$ $+({f}_{3,s}({N}^{\ast })+{p}_{3,s}({N}^{\ast })+m){\int }_{0}^{\infty }{n}_{3,s}(t)dt,$ ${n}_{1,s}(0)=1,\,{n}_{2,s}(0)=0,\,{n}_{3,s}(0)=0$

^1^N* is the final densities of the food web models.

^2^The dynamic equation for larvae stage was modified for these calculations as $\frac{d{n}_{1,s}}{dt}=-{g}_{1,s}({N}^{\ast }){n}_{1,s}-{f}_{1,s}({N}^{\ast }){n}_{1,s}$ −${p}_{1,s}({N}^{\ast }){n}_{1,s}-m{n}_{1,s}$.

The food web model was derived from basic energetic concepts to accommodate the complexity of food webs^26^ (Table 2). The model incorporated size-dependent consumer–resource interactions; energy use for development, survival, and reproduction (energetics); and transitions of individuals among developmental stages (stage-structured populations). Size dependency in consumer-resource interactions is common in aquatic systems^27^. Energy is one of the important currencies for estimating costs for organisms^28^, and life history strategies are shaped by trade-offs between costs for survival and costs for future reproduction^9,29^. Energy is also important in determining parental investment per offspring. The larger the body size of offspring parents produce, the greater the amount of energy they invest in each of their offspring. Recent studies have demonstrated the importance of structured populations in community dynamics^30–32^, and populations in the model need to be structured to accommodate a diversity of life history strategies.

**Table 2:** Dynamic equations and parameters for the food web model.

	Description	Value
**Index Variables**		
*i*	Stage (0: primary producer; 1: larvae; 2: juvenile; 3: adult)	0–3
*s*	Species/Population	1–10
${{\rm{\Omega }}}_{i,s}^{{\rm{predator}}}$	Set of stage and species indices that are predators of stage *i* of species *s*	
${{\rm{\Omega }}}_{i,s}^{prey}$	Set of stage and species indices that are prey of stage *i* of species *s*	
**State Variable**		
*n*_*i,s*_	Density of individuals in a stage *i* of population *s*; Initial value at time 0 is randomly assigned ~Uniform [0,1]	—
*N*	Vector of stage densities	
**Fixed Parameters**		
*m*	Per capita natural mortality rate	0.01
*v*	Coefficient for vulnerability as resource	0.1
*e*	Coefficient for the efficiency of consuming resource	1
*δ*	Efficiency for an individual to convert energy for development and reproduction.	0.5
*μ*	Energy required for maintenance per mass	0.1
*γ*	Efficiency for converting consumed energy to usable energy	0.5
*k*	Carrying capacity of environment for primary producers	100
**Derived Parameters**		
*α*_*i,s*_	Vulnerability to consumption	$v\times {l}_{i,s}^{-1}$
*β*_*i,s*_	Efficiency of consumptions	*e* × *l*_*i,s*_
*w*_*i,s*_	Mass of individuals	${l}_{i,s}^{3}$
**Randomly Assigned Parameters**		
*r*_*s*_	Intrinsic per-capita population growth rate	Uniform [0, 1]
*l*_0*,s*_	Length of primary producers	Uniform [0, 0.5]
*l*_3*,s*_	Length of adults	Uniform [0, 1]
*l*_2*,s*_	Length of juveniles	Uniform [0, *l*_*3,s*_]
*l*_1*,s*_	Length of larvae	Uniform [0, *l*_*2,s*_]
**Dynamic Equations**		**Descriptor**
$\frac{d{n}_{0,s}}{dt}={r}_{s}{n}_{0,s}(1-\frac{{n}_{0,s}}{k})-{p}_{0,s}(N){n}_{0,s}$		Dynamics of primary producers
$\frac{d{n}_{1,s}}{dt}={b}_{s}(N)-{g}_{1,s}(N){n}_{1,s}-{f}_{1,s}(N){n}_{1,s}-{p}_{1,s}(N){n}_{1,s}-m{n}_{1,s}$		Dynamics of larvae
$\frac{d{n}_{2,s}}{dt}={g}_{1,s}(N){n}_{1,s}-{g}_{2,s}(N){n}_{2,s}-{f}_{2,s}(N){n}_{2,s}-{p}_{2,s}(N){n}_{2,s}-m{n}_{2,s}$		Dynamics of juveniles
$\frac{d{n}_{3,s}}{dt}={g}_{2,s}(N){n}_{2,s}-{f}_{3,s}(N){n}_{3,s}-{p}_{3,s}(N){n}_{3,s}-m{n}_{3,s}$		Dynamics of adults
	${p}_{i,s}(N)={\alpha }_{i,s}\sum _{\{i\text{'},s\text{'}\}\in {\Omega }_{s,s}^{predator}}{\beta }_{i\text{'},s\text{'}}{n}_{i\text{'},s\text{'}}$	Predation
	${f}_{i,s}(N)={e}^{0.1\times ({E}_{i,s}-{I}_{i,s})}-1$	Starvation
	${g}_{i,s}(N)=\frac{\delta \times ({I}_{i,s}-{E}_{i,s})}{{w}_{i+1,s}-{w}_{i,s}}$	Development
	${b}_{s}(N)=\frac{\delta \times ({I}_{3,s}-{E}_{3,s})}{{w}_{1,s}}{n}_{3,s}$	Birth
	${I}_{i,s}=\gamma {\beta }_{i,s}\sum _{\{i\text{'},s\text{'}\}\in {\Omega }_{i,s}^{prey}}{\alpha }_{i\text{'},s\text{'}}{w}_{i\text{'},s\text{'}}{n}_{i\text{'},s\text{'}}$	Energy intake
	${E}_{i,s}=\mu {w}_{i,s}$	Energy use

In this study, each food web was initiated with random body size (random physical trait) with some constraints. After solving equations, many animal populations went extinct, and some of the extinct populations were replaced with new animal populations with randomly assigned body size. This process represented the introduction of new species to the food web. Solving equations and replacing animal populations were repeated until at least five animal populations persist in the model. Then, life history traits of animal populations were calculated. The whole process was repeated to obtain 90 independent food webs. Then principal component analysis (PCA) was applied to the life history traits of all animal populations in the 90 food webs. More details of the model and simulation are described in the method section.

## Results

The results from the PCA based on the six life history properties resulted in more than 90% of variation being explained by the first three components (Table 3). The first component (PC1) was strongly influenced by adult duration and generation time. The second component (PC2) had high loadings for percent to maturity, which was negatively associated with both age of maturity and fecundity. The third component (PC3) had high loadings for age of maturity and fecundity (i.e. few large, early maturing offspring vs. many small, late maturing offspring).

**Table 3:** Coefficients on (loadings of) life history properties for principal components.

	% Exp.^(1)^	D. A.^(2)^	G. T.^(3)^	A. M.^(4)^	Fecundity	P. M.^(5)^	L. E.^(6)^
PC1	50.6%	0.550	0.554	0.241	−0.357	−0.019	0.452
PC2	24.0%	−0.126	−0.172	−0.346	−0.248	0.790	0.387
PC3	15.8%	−0.155	−0.059	0.723	0.522	0.296	0.300
PC4	8.9%	0.240	0.175	−0.540	0.706	−0.102	0.333
PC5	0.8%	−0.360	−0.327	−0.019	−0.203	−0.527	0.667
PC6	0.0%	−0.686	0.722	−0.086	−0.006	0.013	−0.009

^(1)^Percent explained, ^(2)^Duration of the adult stage, ^(3)^Generation time, ^(4)^Age of maturation, ^(5)^Percent to maturity, ^(6)^Life expectancy.

**Figure 1:**
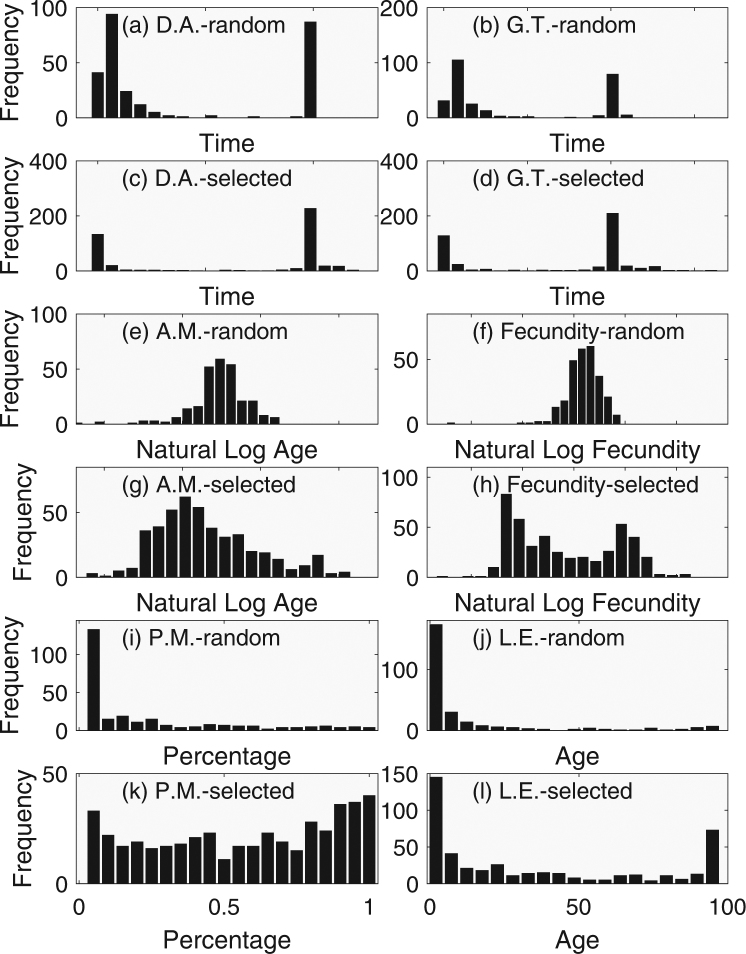
Frequency distributions of life history properties for randomly assembled consumers (prior to simulation) and selected consumers (following simulation). Randomly assembled consumers: (**a**) expected duration of the adult stage (D.A.), (**b**) generation time (G.T.), (**e**) expected age of maturation (A.M.), (**f**) fecundity, (**i**) Percent to maturity (P.M.), and (**j**) life expectancy (L.E.). Selected consumers: (**c**) expected duration of the adult stage (D.A.), (**d**) generation time (G.T.), (**g**) expected age of maturation (A.M.), (**h**) fecundity, (**k**) percent to maturity (P.M.), and (**l**) life expectancy (L.E.).

Two life history traits, the adult duration and generation time, of species with randomly assigned physical traits (body size) exhibited bimodal distributions under food web interactions (Fig. 1a and b), indicating that the food webs were dominated by species that were either long-lived or short-lived, with few species having intermediate life spans. Herein, the former strategy is referred to as iteroparous and the latter semelparous. The bimodal pattern remained even after the food webs were subject to invasion and selection (Fig. 1c and d) although there were more species with greater adult duration, suggesting some level of selection.

The expected age of maturation of species with randomly assigned body size had a unimodal distribution under food web interactions (Fig. 1e). However, the selected species tended to mature younger on average, and the distribution had a greater variation (Fig. 1g). Fecundity also had a unimodal distribution before the selection (Fig. 1f) but exhibited bimodal distribution with a higher variation after the selection (Fig. 1h). According to the result of the PCA, increased age of maturation was associated with increased fecundity (PC3).

Both the percent to maturity and life expectancy tended to be low before selection (Fig. 1i and j), but increased substantially after selection (Fig. 1k and l) although some persisted with low percent to maturity and low life expectancy. According to the principal component analysis, the low percent to maturity was accompanied with increased age of maturity (PC2).

**Figure 2:**
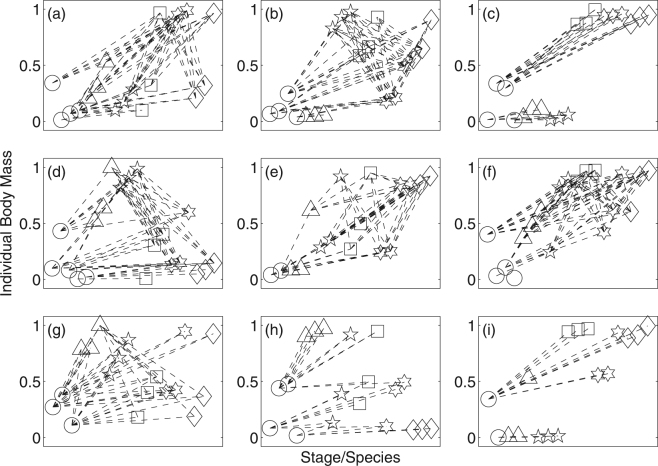
Individual body size (mass) of primary producers and consumers for the first nine food webs. Each circle represents one primary producer species. For consumers (triangles, pentagrams, squares, hexagrams, diamonds), the same symbol was used for the three stages of the same species (larvae, juveniles, and adults from left to right). Dashed lines indicate major consumer resource interactions (interactions between very close trophic levels were omitted to reduce clutter). The vertical axes are for individual body size, and the horizontal axes are for categorical variable for separating different species/stages. The remaining food webs can be found in Supplementary Information (Figures S1–S9).

The body masses of persisting primary producers and consumers in the first nine food webs are plotted in Fig. 2. In most food webs, the number of persisting primary producers was smaller than five. Consumers tend to have either a large or small difference in body size between larvae and adult (i.e. high fecundity with small offspring or low fecundity with large offspring, respectively). This is consistent with the bimodal distribution of fecundity (Fig. 1h). The remaining 81 food webs are shown in Supporting Information (see Supplementary Figs S1–S9). All of these food webs along with the nine shown in Fig. 2 were used in the principal component analysis.

## Discussion

The PCA of life history properties of species that persisted in food web models suggested variations are high in generation time and duration in adult stage (PC1), percent to maturity (PC2), and age of maturation and fecundity (PC3). Understanding the relationships among these life history properties in the food web model are the key to understanding the selection of life history strategies under food web interactions.

Three of the life history properties (percent to maturity, age of maturation, and fecundity) are not independent of each other. The age of maturation was reduced in the model if the larval body size was similar to the adult body size (Fig. 2). This was achieved by producing a small number of large offspring. This occurred because developmental rate was tightly coupled with the body sizes of organisms in the model. Conversely, the age of maturation was increased by producing a large number of small offspring (PC3). Reduced age of maturation was advantageous because it increased the percentage of individuals that reached maturity (PC2). However, increased age of maturation was also advantageous because smaller offspring could utilize different food sources than those used by the adults (less intraspecific competition).

The results of this study suggest that the percent to maturity, age of maturation, and fecundity were selected attributes among life history strategies under consumer–resource interactions because the distributions changed under selection of species. On the other hand, the occurrence of a bimodal pattern among the initial randomly assembled animal, in which a physical trait (body size) was randomly assigned, suggests that the iteroparous–semelparous dichotomy (i.e. duration in adult stage) was the direct result of consumer–resource interactions (Fig. 1a). In other words, it was an imposed life history trait, but not necessarily a selected life history trait because no selection has acted on the randomly assembled species. The iteroparous–semelparous dichotomy resulted because for some species the adult stages were not subject to predation, and so these species lived for an extended period. Other species were subject to greater levels of mortality from predation, and consequently their adult duration was reduced. However, this does not mean that, once the additional mortality from predation is removed, all semelparous organisms would start living longer and reproducing multiple times and persist because the other life history traits (e.g. age of maturation, percent to maturity, and fecundity) are selected. Increased adult duration while maintaining other life history traits would result in over-exploitation of available resource, thus maladapted.

Generation time also exhibited a bimodal distribution before selection (Fig. 1b) because it was strongly affected by duration of the adult stage. However, generation time is also affected by duration of immature stages. Consequently, the distributions for adult duration and generation time were not identical although they were similar.

Finally, life expectancy is a function of the expected age of maturation, percent survival to maturity, and duration of the adult stage. Because the first two were selected under consumer–resource interactions, life expectancy was also selected. Consequently, a greater proportion of species had longer life expectancy than randomly assembled species although many could still persist despite having a short life expectancy (Fig. 1l).

Variation in three of the life history properties (the age of maturation, investment per offspring, and fecundity) have been observed in empirical data^1^. The three key life history traits that emerge under food web interactions in the model also include the same three empirically observed life history properties. Therefore, the model result converges with the empirical observations by Winemiller and Rose^1^, suggesting that the factors incorporated in the model were sufficient to explain the existence of the empirical observed trilateral life history strategies.

The findings in this study may appear contradicting with recent studies demonstrating correlation between physical environmental conditions and life history strategies^22,23,33,34^. However, empirical evidence is still lacking for explaining why different life history strategies co-exist under the same environmental conditions. Importantly, no additional mechanism, such as variation in experienced physical environmental condition, is needed under the current food web model for explaining why animals with various life history strategies can co-exist sharing the same physical environment. Therefore, without dismissing the importance of physical environmental conditions affecting life history strategies, I conclude that species interaction is an important mechanism for the co-existence of species with different life history strategies.

The trilateral life history model of Winemiller and Rose^1^ was based on the analysis of fish life history data. To my knowledge, a similarly clear result has not been demonstrated with terrestrial animals. Therefore, this specific pattern in life history variations may be emphasized more or possibly be unique in the aquatic environment. One of the major differences between the aquatic and terrestrial environments is that species interactions in aquatic food webs tend to be size-dependent more than terrestrial systems^27^. Another important difference is in the frequencies of environmental fluctuations. A frequency tends to be more white (i.e. many frequencies are represented) in the terrestrial environment whereas it tends to be red (i.e. low frequencies are emphasized more) in the aquatic environment^35^. It would be interesting to examine in the future how the patterns in life history strategies change as the model is modified to include size-independent species interactions and/or environmental fluctuations. Then, resulting life history strategies can be compared with those found empirically in the terrestrial environment.

According to Stearns^7^, fitness is “something everyone understands but no one can define precisely”. Consequently, proxies for fitness are often used, for example asymptotic per capita population growth rate^12,36^ or invasion exponents^37^, to investigate the evolution of life history strategies. In the current study, persisting species had the time-averaged instantaneous per-capita population growth rate of approximately zero regardless of their life history strategies; otherwise, they could not persist. Therefore, a population growth rate was not a good measure for the fitness. The invasion exponent^37^ was also not a good measure because the species compositions changed dynamically over time in the model. Species with a certain life history strategy may be successful in invading a particular food web, but not in other food webs. Instead of using these traditional fitness measures, the life history strategies of species that persisted for an extended period of time (2000 time-steps) in food webs were investigated. It was assumed that those species that persisted for a long time had high fitness. Therefore, the fitness is measured by its consequence (persistence) without defining it. This approach was possible because the food web model allowed multiple replications of simulations over a long period, which enabled the statistical analyses of selected life history traits. It should be noted that 2000 time-steps were not long enough for some species to reach the asymptotic dynamics (periodic cycle or stable equilibrium). However, this situation is the same with empirical observations, in which determining whether populations are really under asymptotic dynamics or not is often difficult^38^.

The model used in this study was developed to enable fast simulations of food webs that include multiple structured populations of different species^26^. The model was developed by discretizing stages first and then deriving the approximate rates that relate densities among stages as a function of biomass, with a focus of keeping track of density of individuals. A similar but more sophisticated model with less number of species was also developed by De Roos *et al*.^39^. The De Roos *et al*. model was developed by integrating biomass over age within an immature stage first and then discretizing stages, with a focus of maintaining a consistent total stage-specific biomass of a population. The latter model was successfully used for understanding the dynamics of consumer-resource interactions. For example, it was demonstrated that a stage-structured predator could promote the diversity of its prey, with a bottleneck in the predator’s life cycle reducing predation pressure on some prey that might otherwise have been competitively excluded^31^. Although the two models were developed independently taking different approaches, the fact that the resulting equations are similar to each other is reassuring that both models incorporate the basic factors that govern consumer-resource interactions. For example, the development rate in the former model is a function of difference in biomass between adult and juvenile stages. Simple algebraic manipulations of the maturation term in the De Roos *et al*. model reveals that it is also a function of difference in biomass between mature stage and birth although the size in the latter model is allometrically transformed with an energetic input in the exponent.

The current theories of food web structure and dynamics are conceptually based on communities of unstructured populations. However, recent studies have suggested that changes in niches across developmental stages^30,40^ (ontogenetic niche shifts) and in size structures within a population^31^ may affect food web structure and dynamics. For example, the importance of adjusting the duration of an immature stage to optimize prey availability has previously been reported^3^. These studies primarily investigated the importance of life history strategies on community structure and dynamics. On the other hand, the results from the current study are unique in that they demonstrated the emergence of patterns in life history variations under food web interactions.

Investigating the relationships between species diversity and the properties of ecological communities is an active area of research in ecology. Many recent studies have suggested various factors to stabilize food webs, including body size scaling of feeding relationships^41^, movements of predators over spatially structured prey^42^, the compartmentalization of food webs^43^, and the mixture of strong and weak interactions^44^. The results from this study also suggest that variations in life history strategies are potentially important for their persistence. Then, food web interactions produce life history variations, and the existence of life history variations, in turn, maintain species and species interactions. I suggest life history variations and community dynamics are strongly coupled properties in ecological systems.

## Method

### Food Web Model

The basic structure of the food web model described by Fujiwara^26^ was used in this study except that the number of developmental stages was increased from two to three per consumer population (Table 2). The increased number of stages allowed more potential delays in development, and accommodated more variation in life history strategies. The food web model consisted of a maximum of 10 animal populations, each consisting of three stages: larval, juvenile, and adult stages. They were supported by a maximum of 10 primary producers, which were unstructured populations and exhibited logistic growth. These populations represent different species of animal and primary producer populations.

The occurrence of consumer-resource interactions was determined by body sizes for each potential consumer and resource. In general, a stage with a larger body size consumed a stage of different species with a smaller body size, but a potential resource stage could avoid consumption by being very small or of similar size, relative to the potential consumer. Consumer resource interactions only occurred in the model when the following conditions held: $0.9{w}_{c} > {w}_{r} > 0.125{w}_{c}$, where *w*_*c*_ and *w*_*r*_ are the mass of the consumer and resource, respectively. According to the previous work^26^, which investigated the effects of different relative size ranges for consumer-resource interactions on food web properties, when upper boundary was low, it increased the chance of extinction and reduced the trophic level of consumers. On the other hand, when the lower boundary was high, it increased the trophic level of consumers. Therefore, some intermediate values were selected for this study. No cannibalism was allowed in the current model to eliminate the effects of direct density dependence.

All stages of animal species obtained energy by consuming the primary producers and/or other animal species. The energy gained was used for maintenance and either development or reproduction, depending on whether they were immature (larvae and juveniles) or mature (adults), respectively. The maintenance cost and the body mass of each stage had an allometric relationship. The per-capita developmental rate was proportional to available energy and inversely proportional to the difference in body sizes between an origin stage and destination stage. The per-capita reproductive rate was proportional to available energy and inversely proportional to the body size of larvae. If the energy gain was not sufficient for the body size of a given stage, there was increased mortality from starvation. Each stage of each animal species was subject to three sources of mortality: starvation (as a function of energy gain from consuming, and the body size associated with the corresponding stage); predation (if the body size fitted in the range relative to other stages); and natural mortality (which was incorporated as a constant per capita mortality rate, and included all other causes of mortality).

### Model Simulation and Statistical Analysis

The model simulation was initiated using randomly assigned initial stage densities and body sizes for all animal stages and primary producers. The system of ordinary differential equations (ODEs) was solved from time 0 to 1 (one time-step) using the ODE solver “ode45.m” in MATLAB^45^, based on the default options. Whilst the process of solving the equations, any density <10^−6^ was set to 0 to avoid numerical errors that could cause a population density to become negative. Ecologically, this assumed that there was an Allee effect on populations below the threshold. The system of ODEs was solved repeatedly over 2000 time-steps. At the end of this process, an animal species was considered extinct if the density of any stage was <10^–6^. Similarly, if the density of a plant species was <10^–6^, it was considered extinct. Among the extinct animal species, five were selected and replaced with new species having newly randomly assigned body sizes; this mimicked the introduction/invasion of new species. A half of consumers were replaced with new species. This may represent a high invasion rate, but it ensured a rapid change in species composition to attain stable persistence of multiple consumers. The animal and primary producers that persisted at the end of the previous solutions were assigned the final densities of the previous solutions, and all other species were assigned small (<0.01) random densities. The system of ODEs was solved again over 2000 time-steps, as described above. The processes of solving the ODEs and the introduction of new species were repeated until five animal species persisted in the food web over 2000 time-steps.

After obtaining 90 food webs, each containing five persisting animal species, life history properties were calculated based on the 450 persisting animal species. These properties were: (1) expected duration of the adult stage (adult duration); (2) average age of parents over all offspring (generation time); (3) age of maturation; (4) the percent of offspring that reached maturity (percent to maturity); (5) average fecundity; and (6) life expectancy at birth (longevity) (Table 2). To analyze associations among these properties and their variations, a principal components analysis (PCA) was applied after standardizing data taking z-scores.

The life history properties of randomly assembled species (i.e. prior to simulating the ODEs) were also calculated; however, for the majority of these species, no individual reached the adult stage. Consequently, it was not possible to calculate the generation time and age of maturation. Therefore, only a subset of randomly assembled species for which both generation time and age of maturation could be calculated was included for analysis.
